# Generation of Mouse Small Intestinal Epithelial Cell Lines That Allow the Analysis of Specific Innate Immune Functions

**DOI:** 10.1371/journal.pone.0072700

**Published:** 2013-08-05

**Authors:** Johannes Schwerk, Mario Köster, Hansjörg Hauser, Manfred Rohde, Marcus Fulde, Mathias W. Hornef, Tobias May

**Affiliations:** 1 Department of Gene Regulation and Differentiation, Helmholtz Centre for Infection Research, Braunschweig, Germany; 2 Department of Medical Microbiology, Helmholtz Centre for Infection Research, Braunschweig, Germany; 3 Institute of Microbiology and Hospital Epidemiology, Hannover Medical School, Hannover, Germany; 4 InSCREENeX GmbH, Braunschweig, Germany; McGill University, Canada

## Abstract

Cell lines derived from the small intestine that reflect authentic properties of the originating intestinal epithelium are of high value for studies on mucosal immunology and host microbial homeostasis. A novel immortalization procedure was applied to generate continuously proliferating cell lines from murine E19 embryonic small intestinal tissue. The obtained cell lines form a tight and polarized epithelial cell layer, display characteristic tight junction, microvilli and surface protein expression and generate increasing transepithelial electrical resistance during *in vitro* culture. Significant up-regulation of Cxcl2 and Cxcl5 chemokine expression upon exposure to defined microbial innate immune stimuli and endogenous cytokines is observed. Cell lines were also generated from a transgenic interferon reporter (Mx2-Luciferase) mouse, allowing reporter technology-based quantification of the cellular response to type I and III interferon. Thus, the newly created cell lines mimic properties of the natural epithelium and can be used for diverse studies including testing of the absorption of drug candidates. The reproducibility of the method to create such cell lines from wild type and transgenic mice provides a new tool to study molecular and cellular processes of the epithelial barrier.

## Introduction

Intestinal epithelial cells (IEC) line the mucosal surface along the intestinal tract. They facilitate food degradation as well as nutrient and water absorption and play a critical role in maintaining the host’s metabolic homeostasis. IECs also play a critical role in the absorption of drugs, which are preferably administered via the oral route. The absorption through the intestine is a rather complex and dynamic process, which involves passive diffusion and regulated transport through various influx and efflux drug transporters. In addition, IECs form a tight barrier that separates the gut lumen and the enteric microbiota from the sterile underlying tissue and maintains mucosal immune homeostasis. IECs play an active role in the host microbial interaction and their critical role in mucosal homeostasis and antimicrobial host defence is emerging. Recent studies have demonstrated that intestinal epithelial cells express a variety of innate immune receptors and sense the presence of microbial ligands [1,2]. Ligand exposure leads to the secretion of endogenous mediators and antimicrobial effector molecules. This promotes the recruitment and differentiation of professional immune cells, strengthens the epithelial barrier and provides direct anti-bacterial and anti-viral protection at the site of microbial challenge.

A number of models have been used to characterize the role of IECs in mucosal homeostasis and antimicrobial host defence. Although primary IECs can be prepared with high purity from intestinal tissue, their short survival in culture precludes their use in functional *ex vivo* studies [3–5]. Tumor-derived epithelial cell lines like Caco-2, HT29 or T84 cells have provided an invaluable tool to study the epithelial interaction with microorganisms. In addition, these cell lines improved drug delivery as they helped to elucidate the diffusion and transport processes which occur at the intestinal barrier [6,7]. Human intestinal epithelial tumors, however, are almost exclusively found in the colon and tumor-derived cell lines exhibit a number of ill-defined genetic alterations acquired during cancerogenesis that may significantly alter their function. Stable intestinal epithelial cell lines have also been generated from transgenic mice carrying the SV40 large T antigen [8]. Although these cell lines display many features of IECs, this approach cannot be used to develop cell lines with similar properties from other mouse strains like gene-deficient or transgenic mice. This, however, would be of major interest as genetic approaches to generate epithelium-specific gene-deficient animals have been developed and shown to facilitate important insight in epithelial function *in vivo* [9,10]. The recent discovery of crypt-derived primary organ culture allows for the first time the analysis of viable primary epithelial cells from variable sources [11]. Unfortunately, organoid cultures are both, time-consuming and expensive, and impractical for large-scale analyses. Therefore, a reproducible and flexible approach, which allows the establishment of IEC cell lines from wild type, transgenic or knock out animals with modest financial and time resources is favourable.

The present study describes the generation of stable differentiated and polarized epithelial cell lines by viral transfer of a defined set of immortalizing genes. Both, functional and structural features of the established cell lines are demonstrated by immunofluorescence, electron microscopy and stimulation with exogenous and endogenous immunostimulatory molecules. As proof of principle, the presented method was also applied to genetically modified interferon (IFN) reporter mice [12] to extend the functional value of the established cell lines. The validity of this approach was underlined by the fact that the epithelial phenotype as well as the functionality of the reporter construct was preserved in these cell lines. We were able to establish IFN-sensing reporter IEC cell lines that recapitulate many functional features of the intestinal epithelium *in vitro*. This novel approach provides a versatile tool to generate stable differentiated epithelial cell lines from all types of genetically modified animals for functional and structural analyses.

## Results

### Establishment of epithelial cell lines from fetal mouse small intestinal tissue

For the establishment of novel murine small intestinal epithelial cell lines we used primary cells from fetal tissue for two reasons: First, fetal cells display a stronger intrinsic survival capacity compared to their adult counterparts. Therefore, epithelial cell isolation provides better target cell quality, making the gene transduction process more efficient. Second, fetal cells have not yet been confronted with the gut flora. This allows the establishment of naïve and sterile intestinal epithelial cell lines.

**Figure 1:**
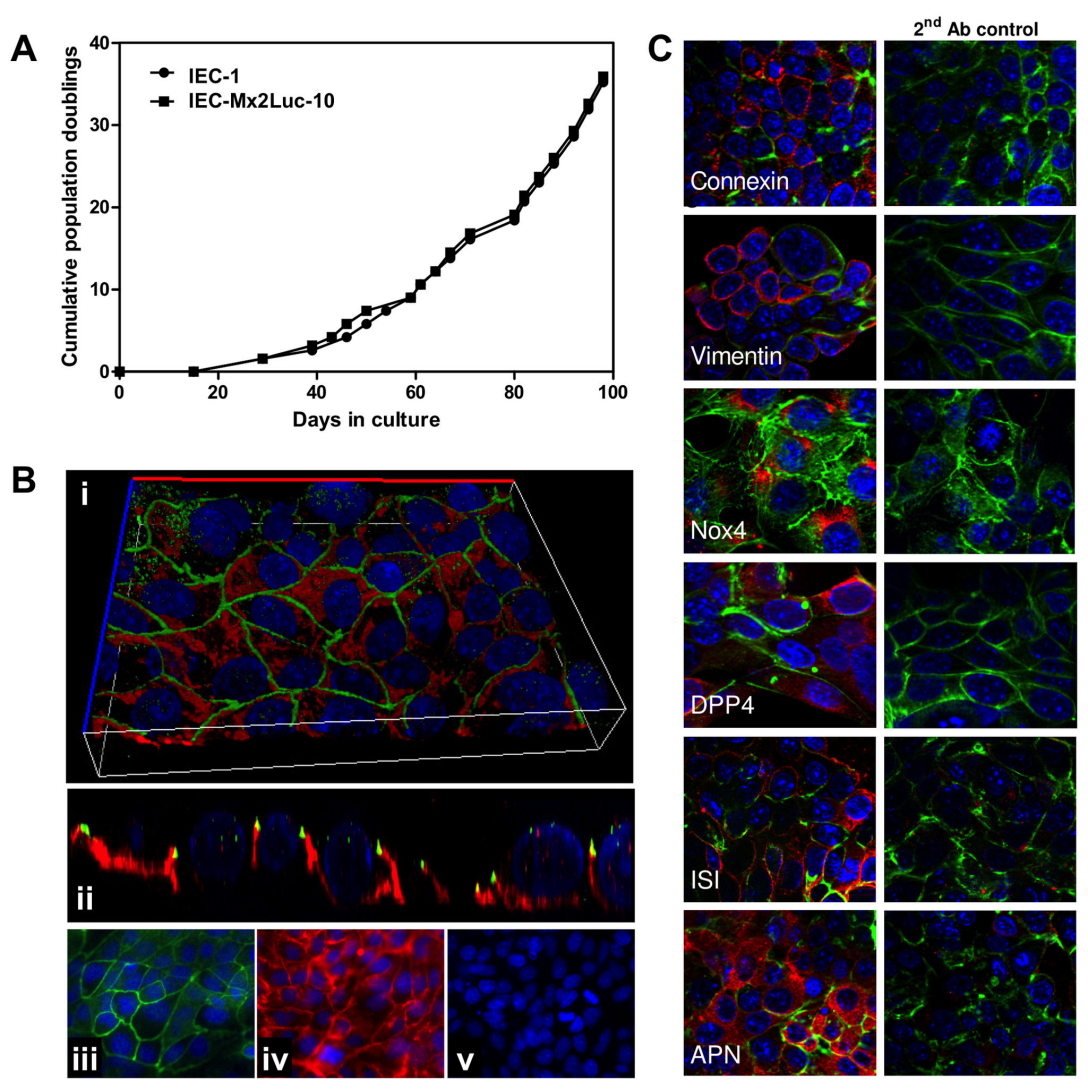
Characterization of polarized growth and surface marker expression. (**A**) Cumulative population doublings of the representative wild type (IEC-1) and IEC Mx2Luc-10 cell clones. Primary cells ceased proliferation, died after approximately 20 days and are not included in the graph. (**B**) Staining of ZO-1 (green) and E-cadherin (red) by immunofluorescence. The image shows a lateral (y/z) view (i) and a three-dimensional reconstruction (ii) of the polarized epithelial cell layer with typical apical intercellular net-like ZO-1 localization and basolateral E-cadherin localization. Specificity of the staining is demonstrated by single channel images for ZO-1 (iii), E-cadherin (iv) and the secondary antibody control (v). Counterstaining with DAPI; magnification x 630. (**D**) Immunostaining for connexin 43, the intermediate filament vimentin, NADPH oxidase 4 (Nox4), dipeptidyl peptidase IV (DPP4), sucrase isomaltase (ISI), aminopeptidase N (APN) in red. The secondary antibody control is included to demonstrate specificity of the staining. Counterstaining with Phalloidin (green) and DAPI (blue); magnification x 630.

Epithelial cells were isolated from small intestinal tissue of mouse E19.5 fetuses. For this purpose we followed the isolation procedure described earlier [13]. After adaption to the culture conditions, isolated cell aggregates were transduced with lentiviruses encoding the genes SV40 large T antigen (TAg), Krüppel-like factor 4 (Klf4), which is a zinc finger transcription factor, and inhibitor of DNA binding 3 (Id3), a dominant negative helix-loop-helix protein. These three factors were identified from a previous screen for factors enabling immortalization of IECs. After transduction, the cells were cultured and maintained in a defined medium. Due to the low intrinsic proliferation potential of primary IECs, a selection procedure based on the growth advantage of positively transduced cells was used. Colonies comprised of proliferating cells became apparent after three weeks. Typically, up to five colonies arose from a 6 well plate and were either picked manually using a light microscope or pooled to establish clonal or polyclonal cell lines, respectively. Fifty to sixty days after transduction, the immortalized cell lines showed a robust proliferation with a doubling time of ~1.3 days (Figure 1A) and a typical cuboid cell shape within contact-dependent growth inhibition and the formation of a cell monolayer (Figure 1B i). This procedure was accomplished for cells derived from wild type and Mx2Luc transgenic IFN reporter mice [12] (see below). The resulting cell lines showed indefinite proliferation without any signs of senescence or crisis for more than 12 months. In total, two polyclonal cell lines and more than twenty clonal cell lines from wild type and Mx2Luc mice were established. Representative monoclonal cell clones were used for further characterization (named IEC-1 and IEC-Mx2Luc-10 respectively). These cell lines could be maintained under standard cell culture conditions with negligible cell death (<5% dead cells). In addition, the cells could be frozen/thawed and subcloned by limiting dilution. Thus, we regard the clonally expanded cells as truly immortalized cell lines.

### Structural and functional characterization to prove the epithelial origin of the established cell lines

The established cell line IEC-1 was further characterized for phenotypic markers to define its cellular origin. Several epithelial marker proteins and functions were determined. Immunofluorescence analysis revealed membrane-bound localization of the junctional protein zonula occludens protein (ZO)-1 and the basolateral tissue adhesion protein E-cadherin (Figure 1B iii, iv). Lateral view analysis and three-dimensional reconstruction of the immunofluorescent images confirmed the typical apical cell border delineating distribution of ZO-1 and the basolateral localization of E-cadherin (Figure 1B i, ii). The cells also stained positive for the gap junction protein connexin 43, the intermediate filament protein vimentin as well as the NADPH oxidase 4 (Nox4), known to be expressed by intestinal epithelial cells (Figure 1C). In addition, expression of three brush border enzymes, which support the metabolic function of the absorptive epithelium, was confirmed by immunofluorescence staining. Expression of all three functional marker proteins, dipeptidyl-peptidase 4 (DPP4), sucrase isomaltase (ISI) and aminopeptidase N (APN) was found on IEC-1 cells (Figure 1C). These data clearly confirm the epithelial origin of the IEC-1 cells and prove that typical features of small intestinal epithelial cells are maintained in the novel immortalized cell line.

**Figure 2:**
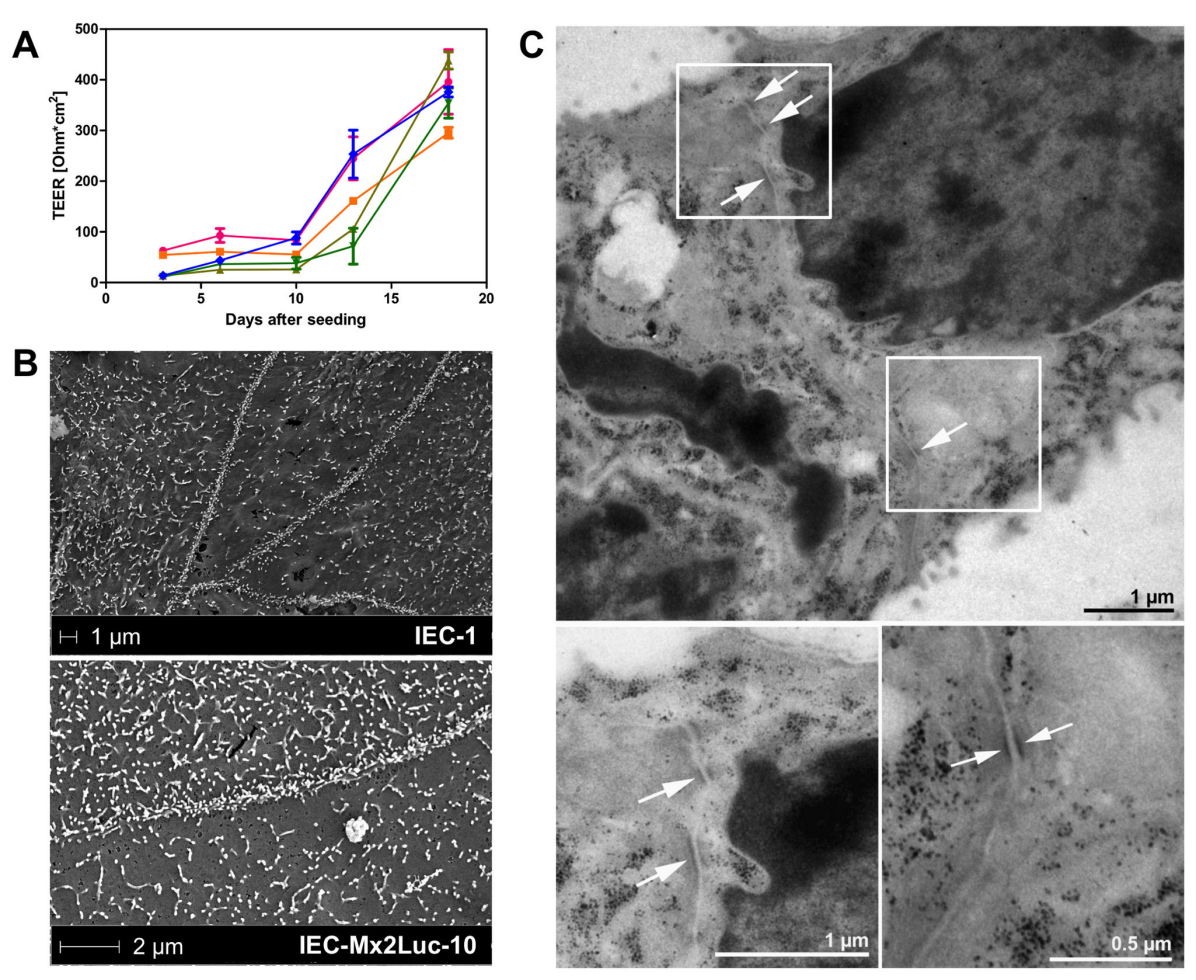
Characterization of epithelial barrier function. (**A**) IEC-Mx2Luc-10 cells were seeded on porous cell culture inserts with a 0.4 µm pore size. Transepithelial electrical resistance (TEER) was monitored over time in triplicates and is expressed as resistance in ohms multiplied by the area of the cell culture insert (Ω*cm^2^ ± SD). (**B**) Electron microscopy of apical microvillus formation 10 days after seeding and (**C**) junctional complex formation (arrowheads) at lateral cell-cell borders 12 days after seeding of IEC-1 and IEC-Mx2Luc-10 cell lines on transwell cell culture inserts.

An important functional feature of the intestinal epithelium is the formation of a tight barrier between the intestinal lumen and the underlying tissue. To assess the barrier formation of the established cell lines, IEC-1 (data not shown) and IEC-Mx2Luc cells were grown on transwell cell culture inserts exposed to cell culture medium at both the basolateral and apical side. The transepithelial electrical resistance (TEER) was measured over time and showed a slow increase over the first five days to values around 100 Ω/cm^2^ followed by a sharp increase to 400 Ω/cm^2^ after fifteen days (Figure 2A). Compared to Caco-2 cells, which are commonly used for *in vitro* absorption and permeability assays and which develop TEER values between 400 and >1000 Ω/cm^2^ [14–17], the TEER obtained from our novel IEC cell lines is slightly lower. This was expected as the IEC cell lines were generated from small intestinal tissue whereas Caco-2 cells are derived from the colon. However, the high TEER values obtained from Caco-2 monolayers are also different from the *in vivo* situation [18], which was also exemplified by *ex vivo* cultures from rats [19].

Finally, ultrastructural features such as microvillus formation and junctional complex formation were detected by electron microscopy. Microvilli represent small apical finger like protrusions that enlarge the absorptive capacity of the intestinal epithelium and enhance nutrient and water absorption at the apical plasma membrane. As expected, IEC-1 and IEC-Mx2Luc-10 cells presented distinctive cell-cell borders and a large number of microvilli at the apical plasma membrane after 10 days of culture on transwell cell culture inserts (Figure 2B). In addition, lateral analysis of cell-cell borders revealed formation of electron-dense junctional complexes 12 days after seeding (Figure 2C). Together, these results illustrate typical functional and structural features of the small intestinal epithelium and clearly identify the potential of the obtained permanently cultured cell lines to grow as polarized small intestinal epithelial cell monolayer with significant differentiation.

### Reactivity towards innate immune receptor stimulation

The critical role of IECs in the antimicrobial host defense became evident in recent years. The underlying mechanisms are incompletely understood but include the stimulation of innate immune receptors followed by the secretion of effector molecules, which in turn limit microbial growth and recruit immune cells to the site of infection.

**Figure 3:**
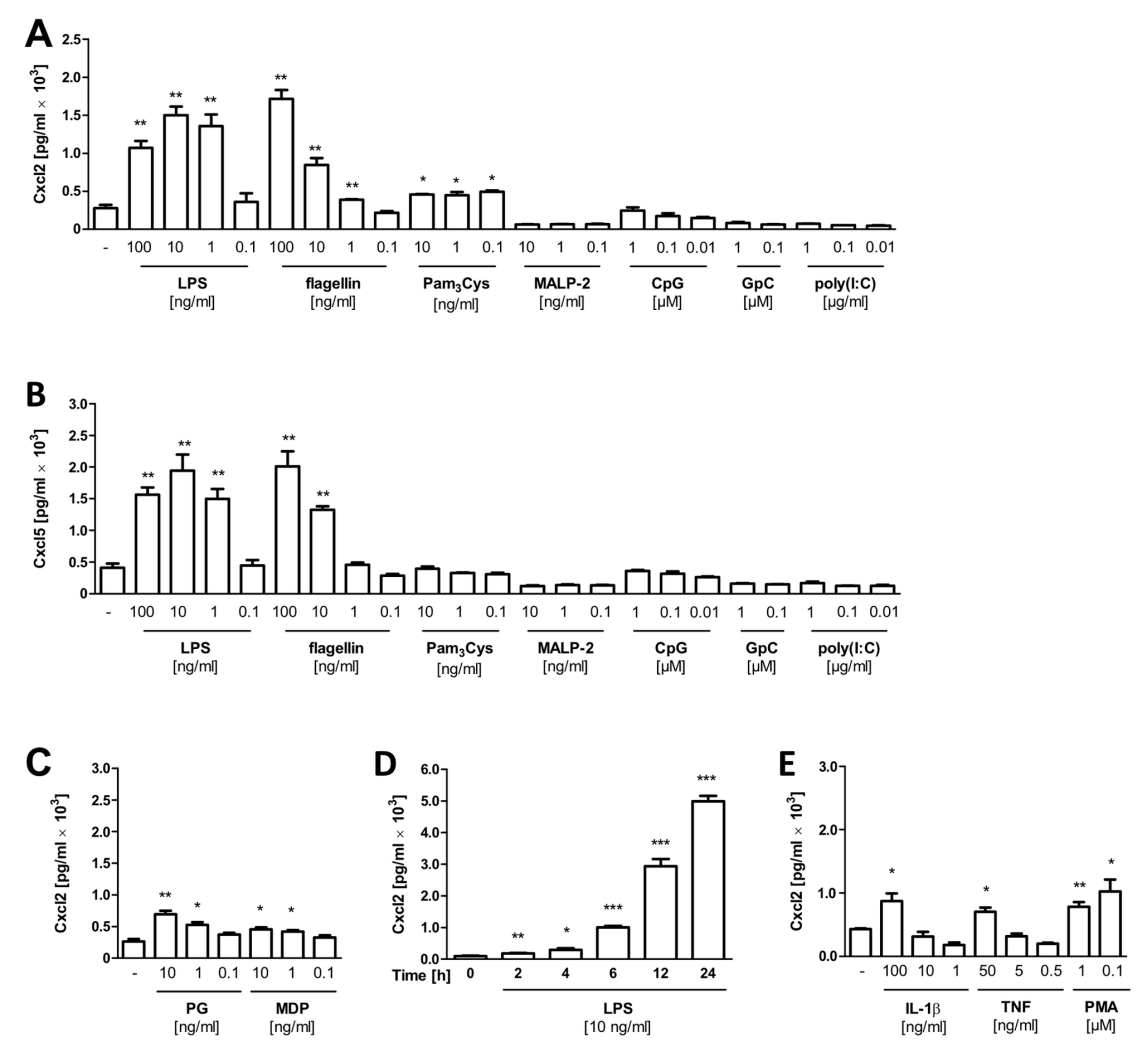
Reactivity towards innate immune receptor stimulation. Cxcl2 (**A**) and Cxcl5 (**B**) secretion in cell culture supernatant measured by ELISA in response to stimulation of IEC-1 cells with the indicated concentration of lipopolysaccharide (LPS), flagellin, the tri-acylated lipopeptide Pam_3_Cys, the di-acylated lipopeptide MALP-2, the CpG oligonucleotide 1668 and its non-stimulatory variant GpC, and poly(I:C) for 6 h at 37° C. (**C**) Cxcl2 secretion in cell culture supernatant measured by ELISA in response to stimulation of IEC-1 cells with the indicated concentration of peptidoglycan (PG) and muramyl di-peptide (MDP) for 6 h at 37° C. (**D**) Kinetics of Cxcl2 secretion after stimulation with 10 ng/ml LPS. (**E**) Cxcl2 secretion in response to stimulation of IEC-1 cells with the indicated concentration of interleukin 1β (IL-1β), tumor necrosis factor (TNF) or phorbol ester (PMA) for 6 h at 37° C. All values are shown as mean ± SD (n=3). Statistics were calculated with the unpaired Student’s *t*-test (*P<0.05, **P<0.01, ***P<0.001).

To elucidate whether the established cell lines mimic this *in vivo* behavior, we examined Cxcl2 and Cxcl5 secretion in the supernatant of IEC-1 cells exposed to various innate immune receptor ligands (also called pathogen associated molecular patterns, PAMPs). A dose-dependent strong increase in the expression of Cxcl2 and Cxcl5 was detected after stimulation with the Toll-like receptor (TLR) 4 and 5 ligands lipopolysaccharide (LPS) and flagellin, respectively (Figure 3A and 3B). In contrast, an only marginal Cxcl2 response was detected towards the TLR2/TLR1 ligand Pam3Cys, a di-acetylated lipopeptide and no reactivity could be observed upon stimulation with the TLR2/TLR6 ligand MALP-2, a tri-acetylated lipopeptide. Similarly, no cellular response was noted after exposure to the TLR3 ligand poly(I:C) and the TLR9 ligand CpG DNA. As expected, the antagonistic TLR9 molecule GpC did not induce epithelial activation. A moderate but significant increase of the Cxcl2 secretion was noted in response to *Staphylococcus aureus* peptidoglycan (PG) and the nucleotide oligomerization domain (Nod) 2 ligand muramyl di-peptide (MDP) (Figure 3C). A time course study illustrated the rapid secretion of Cxcl2 in response to LPS (Figure 3D). Also endogenous immunostimulatory mediators such as interleukin (IL)-1β, tumor necrosis factor (TNF)-α or the unspecific cell stimulus phorbol ester (PMA) enhanced epithelial chemokine secretion (Figure 3E). These results are in accordance with previous studies on the active role of the intestinal epithelium in the host microbial interaction and demonstrate the particular susceptibility of intestinal epithelial cells towards stimulation with TLR4 and TLR5 and Nod2 ligands [1]. Our results highlight the value of the established IEC cell lines, which display the natural physiology of the intestinal epithelium and therefore can serve as suitable *in vitro* models for further investigation of immunomodulatory mechanisms.

### Assessment of interferon reactivity of small intestinal epithelial cell lines

In addition to the wild type IEC-1 cell line we also examined IEC cell lines from a BAC-transgenic interferon (IFN) reporter mouse. The cells of this reporter mouse react very sensitively to type I and type III IFN by expressing luciferase, which is driven by the promoter of the IFN-stimulated gene (ISG) *mx2* [12]. Thus, luciferase production correlates directly with activation of the type I/III IFN signaling pathway and luminometric measurement allows a quantitative determination of the IFN response. The Mx2-Luciferase harboring immortalized cell clones exhibited epithelial properties as described above. A representative cell clone (IEC-Mx2Luc-10) was chosen for further detailed characterization.

**Figure 4:**
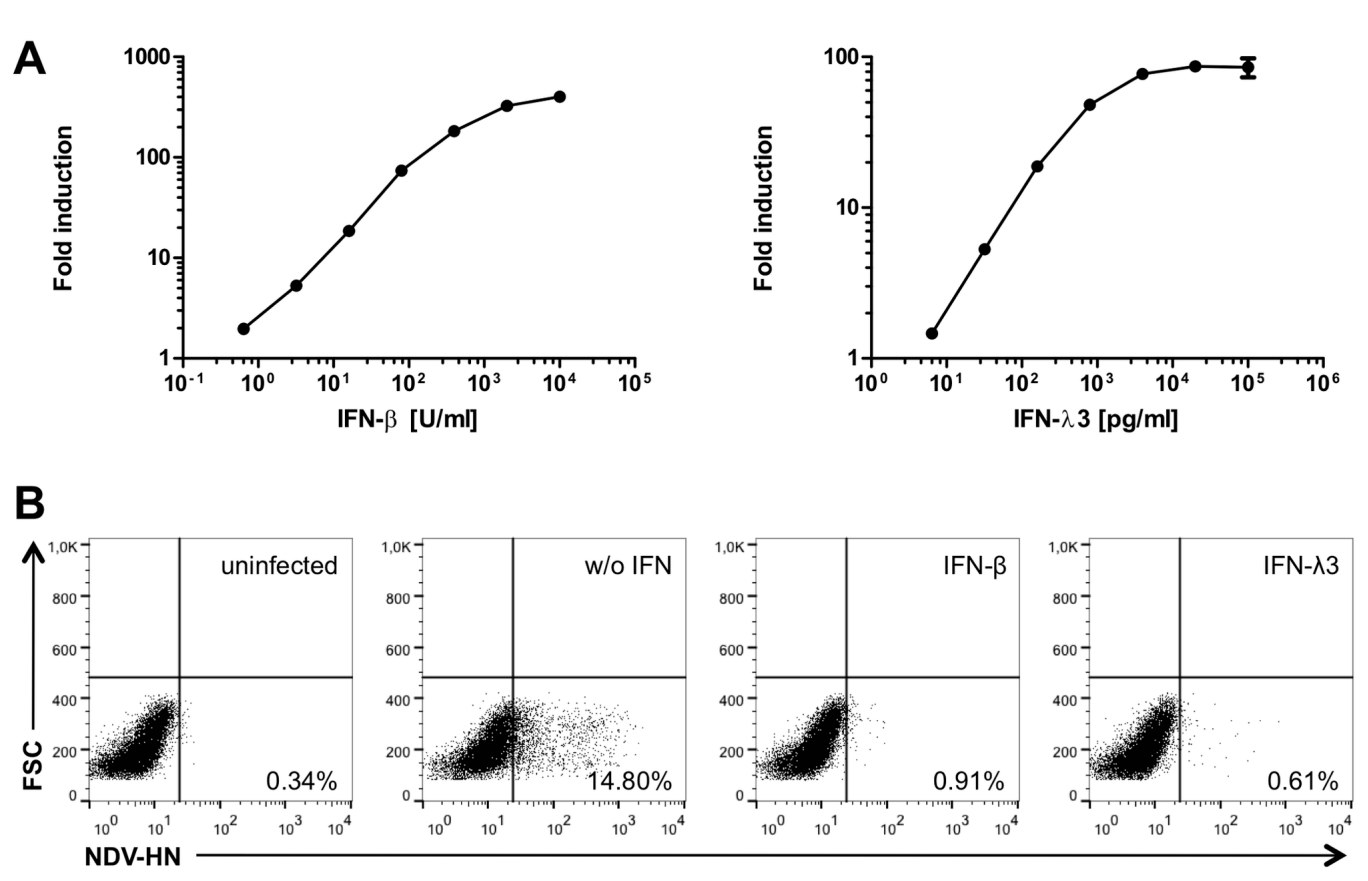
Interferon response of intestinal epithelial interferon reporter cell line. (**A**) Stimulation of IEC-Mx2Luc-10 cells with indicated amounts of IFN-β or IFN-λ3 for 24 h. Fold induction represents relative luminescence units (RLU) of treated compared to untreated cells. All values are depicted as mean ± SD (n=3). (**B**) Antiviral protection of IEC-Mx2Luc-10 cells upon treatment with IFN-β and IFN-λ3. Cells were pre-treated with 1000 U/ml IFN-β or 50 ng/ml IFN-λ3 for 24 h and infected with 80 HAU/ml Newcastle disease virus (NDV) for 24 h. Cells were stained for expression of NDV hemagglutinin-neuraminidase (HN) and analyzed by flow cytometry.

We tested the IEC-Mx2Luc-10 cell clone for its ability to respond to type I and type III IFN. Stimulation with both, IFN-β and IFN-λ3, for 24 h led to strong expression of the Mx2-Luciferase reporter, albeit to a much higher degree with increasing amounts of IFN-β as compared to IFN-λ3 (Figure 4A). Due to the remarkable low basal activity of the *mx2* promoter, the IEC-Mx2Luc-10 cells possess the capacity of a 100-fold induction.

Next, we asked if the induction of the IFN signaling pathway demonstrated by the expression of luciferase also results in antiviral protection of the cells. Therefore, we pre-treated IEC-Mx2Luc-10 cells with IFN-β or IFN-λ3 before infection with Newcastle disease virus (NDV) La Sota strain. The protective effect of IFN against NDV infection has been well characterized [20,21]. IEC-Mx2Luc-10 cells were pre-treated with 1000 U/ml IFN-β or 50 ng/ml IFN-λ3 for 24 h and infected with 80 HAU/ml NDV, approximating a multiplicity of infection (MOI) of 1. The amount of productively infected cells was determined by intracellular staining and flow cytometric analysis of NDV hemagglutinin-neuraminidase (HN) expression 24 h after infection. NDV replication could not be observed in IEC-Mx2Luc-10 cells that were pre-treated with IFN-β or IFN-λ3, whereas 14% of the untreated cells exhibited expression of NDV HN 24 h after infection (Figure 4B). Thus, a protective antiviral state can be induced in IEC-Mx2Luc-10 cells upon pre-treatment with IFN.

### Induction of IFN and ISGs by viral and bacterial pathogens

**Figure 5:**
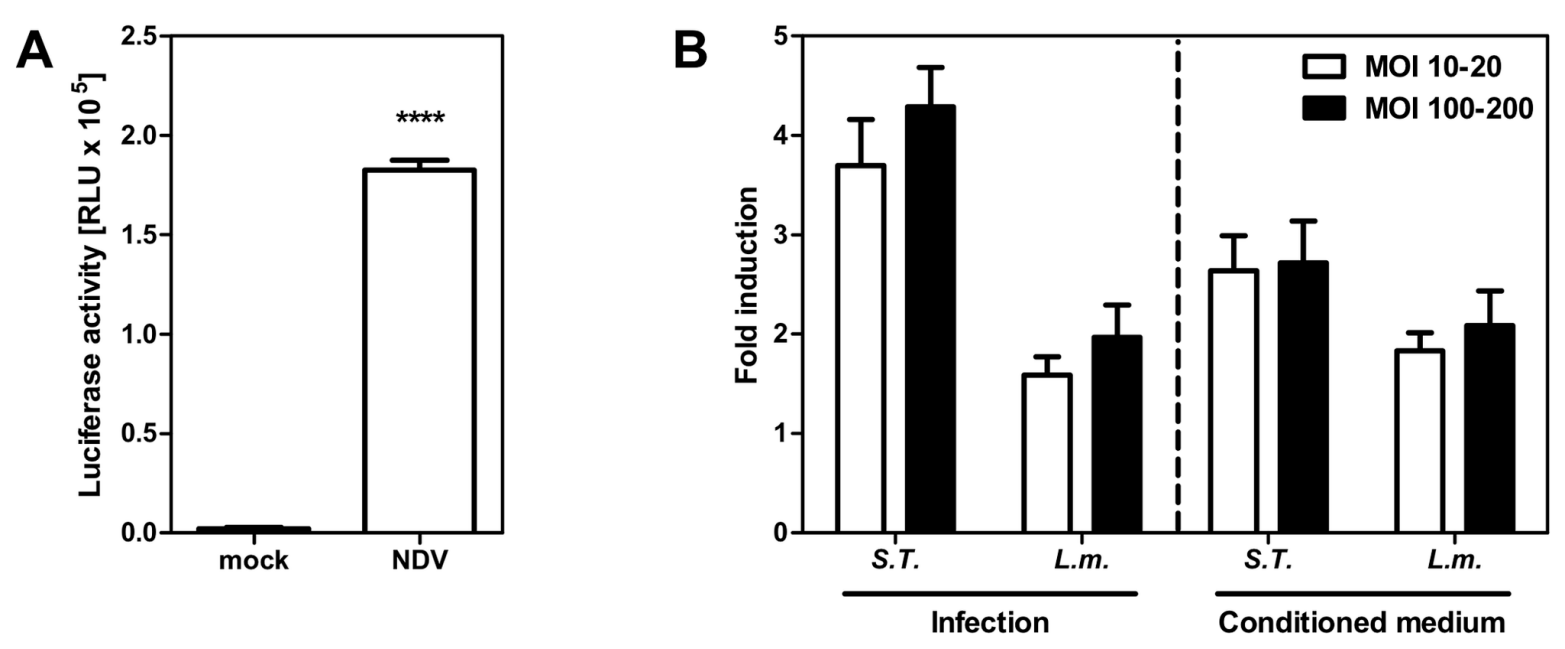
Stimulation of intestinal epithelial interferon reporter cell line by viral and bacterial infection. (**A**) *mx2* induction in IEC-Mx2Luc-10 cells upon infection with 80 HAU/ml Newcastle disease virus (NDV) for 24 h (n=3). (**B**) *mx2* induction in IEC-Mx2Luc-10 cells upon infection with *Salmonella* *Typhimurium* and *Listeria monocytogenes* at a multiplicity of infection (MOI) of 10-20 or 100-200 for 74 h (n=4) and quantification of type I/III IFN secretion upon bacterial infection by incubation of IEC-Mx2Luc-10 cells with filtered conditioned medium from bacterially infected cells for 24 h (n=3). Fold induction represents relative luminescence units (RLU) of infected/stimulated compared to untreated cells. All values are shown as mean ± SD. Statistics were calculated with the unpaired Student’s *t*-test (****P<0.0001).

Type I IFN production is considered as a hallmark of the innate immune response against a diverse range of pathogens [22–24]. We asked if the novel IEC-Mx2Luc-10 cells, besides being good IFN responders, are able to produce IFN. Infection of the cells with NDV, a strong inducer of type I IFN [25], for 24 h elicited a strong IFN response (Figure 5A), proving that the IEC-Mx2Luc-10 cells are also able to produce IFN upon infection.

A considerable proportion of pathogens targeting the intestinal epithelium are invasive bacteria. The role of type I IFN in bacterial infections is controversially discussed [23,24]. There are several studies demonstrating a negative impact of type I IFN during enterobacterial infection [26–28]. However, type I IFN has also been shown to protect mice against *Salmonella* *Typhimurium* and *Listeria monocytogenes* infection [29,30]. Anyhow, IFN is involved in immunity against intracellular bacteria. Therefore, we tested the IEC-Mx2Luc-10 reporter cells for their ability to respond to both bacterial pathogens. Cells infected with *S* *. Typhimurium* demonstrated a 4-fold induction of the *mx2* promoter 74 h after infection (Figure 5B). We found a similar induction, however to a lesser extent, after infection with *L. monocytogenes*. The fold induction of the IFN signaling pathway by bacterial pathogens was considerably lower compared to the induction by viral pathogens (4 fold versus 100 fold). Interestingly, *mx2* induction was not significantly different after infection with low (MOI 10-20) and high (MOI 100-200) amounts of both bacteria. In order to analyze the impact of secreted IFN in the cellular response to bacteria, IEC-Mx2Luc-10 cells were incubated for 24 h with conditioned medium obtained from cells infected for 72 h with *S* *. Typhimurium* and *L. monocytogenes*. The reporter cells responded readily to conditioned medium, demonstrating that ISG expression upon bacterial infection was induced by soluble IFN, secreted by the cells. Comparison of the luciferase activity with the dose response curve (see Figure 4A) revealed that the IFN response elicited by *S* *. Typhimurium* infection equates to 5 U/ml IFN-β.

Taken together, these data demonstrate that the newly established transgenic IEC-Mx2Luc-10 cell line presents a suitable tool for elucidation of the complex mechanisms underlying IFN expression and the innate immune response of the small intestinal epithelium.

## Discussion

A major limitation in basic and applied research is the availability of physiologically relevant *in vitro* models. For *in vitro* studies, isolated primary cells and immortalized cell lines are currently the two major possibilities. Cell lines are readily available, can be easily maintained and genetically modified, but frequently show major structural or functional alterations as compared to their *in vivo* counterparts. Primary cells, on the other hand, closely reflect the *in vivo* situation but are of restricted availability and homogeneity. In particular, human primary cells show a high degree of donor-to-donor variability [31]. *In vitro* models for small intestinal epithelial cells are highly preferable since the intestinal epithelium plays a critical role in mucosal immunology and host microbial homeostasis [1]. The same applies for drug development studies that aim to predict the absorption properties and thus pharmacokinetic features of novel drug candidates correctly [32].

We developed a novel approach to generate small intestinal epithelial cell lines and demonstrate that these cell lines can be maintained like standard cell lines regarding subculturing, freeze/thaw procedures and genetic modification (data not shown). In addition, these cells display specific marker proteins for the intestinal epithelium and retain the ability to develop a functional epithelial cell barrier. This function is a critical parameter in the development of novel drug candidates, as the absorption of chemical compounds is key when the drug is given orally. This can be achieved via passive processes like diffusion or via the paracellular route, but also through active transport processes through ATP-dependent transporters [33]. Our results show that the novel IEC cell lines develop a functional barrier within two weeks if they are cultivated on transwell cell culture inserts. Such a functional barrier allows the monitoring of passive diffusion processes of chemical substances. Whether the novel IEC cell lines are suited for studies that focus on active transport processes or metabolism remains to be investigated and depends on the presence of the respective transporters and/or enzymes. In contrast to Caco-2 cells, a cell line commonly used to investigate epithelial absorption processes, our established cell lines are derived from the small intestine – the main site for absorption *in vivo*. Additional experiments are required to reveal the potential value of the presented cells for investigation of passive and active absorption processes.

An important aspect of this study is that the development of novel IEC cell lines is mediated by a combination of lentiviral transduction with several immortalizing genes. This allows flexibility and reproducibility, and facilitates the development of murine intestinal epithelial cell lines from virtually any mouse genotype. In particular, this approach can be deployed to generate IEC cell lines from knock out, knock in or transgenic mice, which in turn can be used to decipher the molecular mechanisms underlying crucial aspects of intestinal epithelial biology. It remains to be seen if this approach can be translated to epithelial cells from other sections of the gastrointestinal tract or to epithelial cells from adult mice. Moreover, we can only speculate whether this protocol is also valid for the establishment of human intestinal epithelial cell lines. The immortalization of primary human cells requires the modulation of more signaling pathways as compared to their murine counterparts [34]. Therefore, it is likely that the three factors used in this study are not sufficient to immortalize human intestinal epithelial cells and that additional factors are required.

An essential function of IECs is the mediation of innate immunity against intestinal pathogens. Therefore, we focused on the type I and III IFN-induced signaling pathway, which is a prominent player in intestinal innate immunity [35–38] and homeostasis [39,40]. For this purpose, we generated IEC cell lines from a BAC transgenic IFN reporter mouse which reacts sensitively to type I and III IFN [12,41] and allows quantification of IFN signaling by measurement of luciferase activity. An earlier study showed that immortalization via SV40 large T antigen (TAg) only leads to alterations in IFN signaling [42]. This is not the case for the transgenic IEC-Mx2Luc IFN reporter cell lines that were developed in this study. In fact, these cells display responsiveness to IFN and viruses as previous *in vivo* studies indicated for type I or III IFN stimulation and rotavirus infection [12,36]. The reason for this discrepancy is not fully understood but it is likely that immortalization, which relies on the TAg only, requires a high expression level of the viral oncogene. TAg interferes with hundreds of cellular genes [43] and thus, high levels of TAg expression might also affect IFN signaling. In the study presented here, TAg acts in collaboration with other genes (Id3 and Klf4) to establish the IEC cell lines. We hypothesize that a low expression level of TAg together with the action of the other genes leads to functional immortalization.

Taken together, the IEC-Mx2Luc cells are a suitable tool for detection of low-level, nuanced type I and type III IFN responses that recapitulate the *in vivo* situation and to study the molecular mechanisms of innate immunity of the small intestinal epithelium. Although it is known that microbial products initiate signal transduction and secretion of effector molecules within intestinal epithelial cells, little is known about effector molecules and regulatory mechanisms governing the innate immune response. In order to understand these complex mechanisms, various mouse models have been established. Most notably, we could demonstrate that an important feature, which is the barrier function, is functional in the immortalized IECs. Since barrier integrity is dramatically influenced by inflammatory stimuli and its loss is associated with reduction of anti-pathogenic activity, intact barrier function is a prerequisite when epithelial cell lines are deployed as an *in vitro* model to study innate immune reactions. The study presented here allows easy generation of relevant IEC cell systems to study molecular and cellular processes of the epithelial barrier.

## Materials and Methods

### Mice and ethics statement

All mice were bred under standard conditions at the Helmholtz Centre for Infection Research (Braunschweig, Germany). Animals were handled in strict accordance with good animal practice as defined by the relevant local animal welfare bodies, and all animal work was approved by the appropriate committee (Niedersächsisches Landesamt für Verbraucherschutz und Lebensmittelsicherheit [LAVES], Oldenburg, Germany).

### Cell cultivation

Primary intestinal epithelial cells were isolated as described recently [13]. Briefly, the epithelial cell layer of E19 fetal small intestinal tissue was detached from the underlying tissue of inverted intestinal segments by incubation in 30 mM EDTA and separated from the underlying tissue using a cell strainer. The primary organoid cultures were cultivated on collagen-coated 12 well plates in a humidified atmosphere with 5% CO_2_ at 37° C. Cells were maintained in a defined IEC medium (InSCREENeX, Braunschweig, Germany) and transduced with self-inactivating lentiviral vectors of the third-generation [44]. The expression cassettes were implemented into a previously described lentiviral vector in which the 5’-HIV U5 element was replaced by the RSV promoter [45]. In addition, a cPPT element was inserted 5’ and the Hepatitis B post-transcriptional regulatory element (PRE) was inserted 3’ to the expression cassette in order to enhance expression of the transgenes. Three different lentiviral vectors were employed encoding for (a) SV40 large T antigen, (b) Klf4 and (c) Id3 (sequences are available on request). The expression of these recombinant genes is driven by the SV40 promoter. For transduction, the abovementioned lentiviral vectors were simultaneously incubated with the primary organoid cultures over night in the presence of 8 µg/ml polybrene. Culture medium was renewed the next day and cells were maintained until proliferating colonies arose, which was typically three weeks after transduction. During this time the medium was renewed every third day. Using this approach, about 1-2 immortalized colonies were established per 1x10^5^ primary cells, which were picked manually and clonally expanded or cultivated as polyclonal cell pools. The resulting cell lines were expanded using splitting ratios between 1:5 and 1:10. The phenotypic characterization of the cell lines was performed between cumulative population doublings 15 and 40.

### Measurement of transepithelial electrical resistance

Measurement of the TEER was performed as described previously [46]. Briefly, 1x10^5^ cells were seeded on porous transwell cell culture inserts (Costar) with a pore size of 0.4 µm. The TEER was measured with a Voltohmmeter (EVOM, World Precision Instruments) for a period of 20 days with renewal of the culture medium every third day. Transwell inserts without cells were used to determine the basal resistance of the membrane in the medium. Measurements were performed in triplicates for each time-point. The TEER is expressed as the measured resistance in ohms multiplied by the area of the transwell insert (Ω*cm^2^).

### Phenotypic characterization

Immunofluorescence staining of ZO-1 and E-cadherin was performed using a rabbit anti-ZO-1 antiserum and a mouse monoclonal IgG2a anti-E-cadherin antibody (purchased from Zymed Laboratories and BD Transduction Laboratories, respectively) followed by the appropriate FITC- and Cy3-conjugated secondary antibodies (Jackson ImmunoResearch Laboratories). The rabbit anti-connexin 43 antibody was purchased from Cell Signalling Technology (Danvers, MA) and the goat anti-vimentin antiserum from Sigma (Taufenkirchen, Germany). The NADPH oxidase 4 (Nox4), dipeptidyl peptidase IV (DPP4), sucrase isomaltase (ISI) and aminopeptidase N (APN) rabbit antisera were generously obtained from Alain Vandewalle (University X. Bichat, Paris, France) and used in combination with a Cy3-conjugated anti-rabbit secondary antibody (Jackson ImmunoResearch Laboratories). Actin staining was performed by incubation with FITC-Phalloidin 1:200 (Sigma-Aldrich) for 10 min. DAPI containing vectashield-mounted slides (Vector Laboratories) were analyzed using an ApoTome-equipped Axioplan 2 microscope connected to an AxioCam Mr digital camera (Carl Zeiss MicroImaging, Inc.). Three dimensional image reconstruction was performed using the Axiovision software from Zeiss (Jena, Germany).

### Electron microscopy 

Cells grown on collagen-coated 0.4 µm pore size transwell inserts were fixed with 2% glutaraldehyde/5% formaldehyde in HEPES buffer containing 10 mM MgCl_2_, 10 mM CaCl_2_ and 0.09M sucrose, pH 6.9, for 1 h on ice, washed with buffer, and further fixed with 1% aqueous osmium for 1 h at room temperature. Samples were then dehydrated with a graded series of acetone (10%, 30%, 50%). At the 70% dehydration step samples were left over night in 70% acetone containing 2% uranylacetate, and further dehydrated with 90% and 100% acetone. Samples were then embedded in the epoxy resin Spurr (hard formular) according to described procedures [47]. Ultrathin sections were cut with a diamond knife, picked up with butvar-coated grids, counterstained with uranyl acetate and lead citrate, and examined in a TEM 910 transmission electron microscope (Zeiss, Oberkochen, Germany) at an acceleration voltage of 80 kV. Images were recorded digitally at calibrated magnifications with a Slow-Scan CCD-Camera (ProScan, 1024x1024, Scheuring, Germany) with ITEM-Software (Olympus Soft Imaging Solutions, Münster, Germany).

For scanning electron microscopy, fixed cells grown on cell culture inserts were dehydrated with a graded series of ethanol, critical point dried with CO_2_ and sputter coated with palladium–gold. Samples were examined in a Zeiss (Oberkochen, Germany) Merlin Field emission scanning electron microscope at an acceleration voltage of 5 kV. The secondary electron (SE) signal was detected with the Everhart-Thornley and inlens SE-detector in a 50:50 ratio.

### ELISA assays 

For stimulation experiments, cells were grown in 48 well plates for 4 days and the indicated stimulus was added in triplicates to the cell medium. *E. coli* Dm31 LPS was purchased from List Biological Laboratories (Denver, CO), Pam _3_Cys from Enzo Life Sciences (Lörrach, Germany), MALP-2 from Alexis Biochemicals (Lausen, Switzerland), stimulatory CpG Oligo 1668 with the sequence 5’ TCCATGACGTTCCTGATGCT3’ and non-stimulatory GpC oligo with the sequence 5’ TCCATGAGCTTCCTGATG3’ from Eurofins MWG Operon (Ebersberg, Germany), flagellin from InvivoGen (San Diego, CA), endotoxin-free recombinant mouse IL-1β and TNF from eBioscience (Frankfurt, Germany), poly(I:C) (P1530), peptidoglycan (PG, isolated from *S. aureus*), synthetic muramyl di-peptide (MDP), and phorbol ester (PMA) were all purchased from Sigma. After the indicated incubation period, the cell culture supernatant was harvested and stored at -20° C until further analysis. The chemokines Cxcl2 and Cxcl5 were quantified in cell culture supernatant using a commercial ELISA from Nordic Biosite (Täby, Sweden).

### Source of interferon and stimulation 

Murine IFN-β was recombinantly produced in stably expressing BHK-21 cells and obtained from cell culture supernatant as described before [12]. Murine IFN-λ3 was purchased from PBL Interferonsource (Piscataway, NJ). For stimulation experiments, cells were grown to 70-90% confluency and stimulated with indicated concentrations of IFN (diluted in medium) for the indicated time.

### Measurement of luciferase activity

For quantification of the enzymatic activity of luciferase, cells were lysed in reporter lysis buffer (Promega, Madison, WI). Cell extracts were assayed for luciferase activity using a standard reaction buffer (20 mM glycylglycine, 12 mM MgSO_4_, 1 mM ATP) containing luciferin (Synchem, Felsberg/Altenburg, Germany) and a single-tube luminometer (Berthold, Bad Wildbad, Germany).

### Bacterial infection of cells

*Salmonella* *Typhimurium* and *Listeria monocytogenes* (both a kind gift of Siegfried Weiss, HZI) were grown in Luria-Bertani medium (LB) for *S* *. Typhimurium* ) or brain heart infusion medium (BHI) for *L. monocytogenes*, respectively, at 37° C. Cells grown to 70-90% confluency were infected with *S* *. Typhimurium* and *L. monocytogenes* at the indicated multiplicities of infection (MOI) following standard protocols. Briefly, bacteria grown to the early stationary phase were washed twice in PBS and diluted in culture medium to the indicated MOI. Cells were infected with the inoculum for the indicated time. Extracellular bacteria were removed by three times washing and addition of 50 µg/ml gentamicin to the medium 1 h after infection, and an additional washing step followed by a decreased gentamicin concentration of 10 µg/ml 2 h after infection. Experiments were performed in triplicates or quadruplicates.

### Quantification of antiviral protection

Cells were pre-treated with IFN-β or IFN-λ3 for 24 h. Infection with Newcastle disease virus (NDV) La Sota (Lohmann Tierzucht, Cuxhaven, Germany) was performed after three times washing with serum-free medium. 1 h after infection, residual virus was removed by washing three times with serum-containing medium. 24 h after infection, cells were fixed in suspension with 4% paraformaldehyde (10 min) followed by Triton X-100 (0.1%) permeabilization (10 min). Primary mouse anti-HN14f antibody (Santa Cruz) was used at 1:500. Secondary Cy5-labelled goat anti-mouse IgG + IgM antibody (Dianova, Hamburg, Germany) was used. Flow cytometric analysis of fixed cells was performed with a BD FACS-Calibur, using appropriate laser and filter settings for Cy5. Results were quantified with FlowJo 7.6 software.
